# AMFormer-based framework for accident responsibility attribution: Interpretable analysis with traffic accident features

**DOI:** 10.1371/journal.pone.0329107

**Published:** 2025-07-28

**Authors:** Yahui Wang, Zhoushuo Liang, Yue He, Jiahao Wu, Pengfei Tian, Zhicheng Ling

**Affiliations:** 1 School of Medical Technology, Beijing Institute of Technology, Beijing, China; 2 School of Computer Science, South-Central Minzu University, Wuhan, Hubei, China; 3 School of Software, Liaoning Technical University, Huludao, Liaoning, China; 4 School of Humanities and Management, Wannan Medical College, Wuhu, Anhui, China; Southwest Jiaotong University, CHINA

## Abstract

Accurately determining responsibility in traffic accidents is crucial for ensuring fairness in law enforcement and optimizing responsibility standards. Traditional methods predominantly rely on subjective judgments, such as eyewitness testimonies and police investigations, which can introduce biases and lack objectivity. To address these limitations, we propose the AMFormer(Arithmetic Feature Interaction Transformer) framework—a deep learning model designed for robust and interpretable traffic accident responsibility prediction. By capturing complex interactions among key factors through spatiotemporal feature modeling, this framework facilitates precise multi-label classification of accident responsibility. Furthermore, we employ SHAP (SHapley Additive Interpretation) analysis to improve transparency by identifying the most influential features in attribution of responsibility, and provide an in-depth analysis of key features and how they combine to significantly influence attribution of responsibility. Experiments conducted on real-world datasets demonstrate that AMFormer outperforms both other deep learning models and traditional approaches, achieving an accuracy of 93.46% and an F1-Score of 93%. This framework not only enhances the credibility of traffic accident responsibility attribution but also establishes a foundation for future research into autonomous vehicle responsibility.

## 1. Introduction

As vehicle numbers rise and road traffic grows more complex, traffic accidents have become more frequent, worsening road safety [1]. This presents significant challenges for traffic safety management [2]. According to laws and regulations, handling traffic accidents involves on-site investigation, responsibility determination, punishment, compensation, and mediation. Among these, determining responsibility is critical. Traffic accidents are closely tied to the interests of those involved, and responsibility decisions affect their rights, social fairness, and law enforcement credibility [3]. Therefore, developing an accurate, reliable, and interpretable model for assigning responsibility is essential. Traffic accident responsibility attribution refers to the fault behaviors of the parties involved and their responsibilities. It involves both qualitative and quantitative descriptions of the causes, as well as the classification and quantification of legal violations [4]. This is vital for determining the nature and severity of penalties [5]. Responsibility levels can be categorized into full, no, primary, secondary, or equal responsibility based on collision records [6]. Analyzing the influence of factors under different responsibility levels helps make attribution results more transparent and convincing. Despite progress, challenges remain in identifying responsibility for traffic accidents. One issue is that traffic accident personnel may lack sufficient knowledge in law and technology, leading to reliance on empirical judgment, which can compromise objectivity and scientific accuracy [7]. Additionally, while some research focuses on identifying primary and secondary liabilities, accidents often involve multiple factors, such as weather, road conditions, vehicle types, and driver states [8]. Current methods may be limited when dealing with complex, dynamic traffic accident scenarios. For both drivers and decision-makers, understanding the rationale behind responsibility division is critical, but existing approaches often lack adequate explanatory support [9]. Although some studies address specific risk factors, they typically view accident risk as stemming from a single source, ignoring the interplay of multiple factors. While research on combined risk factors is emerging, these studies often adopt a “black-box” approach [10,11], which lacks transparency and interpretability, making it difficult for accident parties to trust the results. The motivation behind this research is to address these issues by providing a more comprehensive, transparent, and interpretable model for accident responsibility prediction. To this end, this study proposes a deep learning-based framework for traffic accident responsibility prediction and attribution. The framework utilizes the AMFormer model to predict responsibility levels in traffic accidents. Unlike traditional methods, which often rely on a limited set of factors [12], our framework incorporates a broader approach, accounting for various contributing factors. Based on the responsibility labels in vehicle collision records, the driver’s degree of responsibility is categorized into full responsibility, no responsibility, primary responsibility, secondary responsibility, and equal responsibility. Given the complexity of these categories and the various factors influencing responsibility, it is important to understand how each factor contributes to the determination of responsibility. To address this, the study incorporates an explainability analysis module based on SHAP. This module provides detailed interpretability for each responsibility category (full responsibility, no responsibility, primary responsibility, secondary responsibility, and equal responsibility), investigating how different factors contribute to each level of responsibility. Through this approach, we explore the varying impact of factors such as weather, road conditions, vehicle types, and driver behavior on the determination of responsibility.

The main contributions of this study are as follows: (1) A deep learning model based on AMFormer is applied to predict the classification of driver responsibility levels in traffic accidents. (2) Shapley values are used to perform a multi-class interpretability analysis for different responsibility levels. The study examines the impact of different types of influencing factors on responsibility attribution when the driver responsibility levels are categorized as full responsibility, no responsibility, primary responsibility, secondary responsibility, or equal responsibility. This study contributes to improving the accuracy and interpretability of accident responsibility attribution, making the attribution process more transparent and enabling a clearer understanding of the causes of accidents. This, in turn, provides a foundation for more effective preventative measures, ultimately reducing the occurrence of traffic accidents.

## 2. Related works

In this section, we first discuss the relevant theories of accident responsibility attribution. Subsequently, we review the current research on factors influencing driver violations. Lastly, we present the criteria and standards currently used to determine accident responsibility.

### 2.1. Accident culpability attribution

Accident responsibility prediction and attribution have been a key focus in traffic safety research. Accidents result from multiple human and technical factors. Fritz Heider highlighted human factors such as operational errors, negligence, and violations of safety procedures, influenced by cognitive biases, fatigue, or inadequate skills [13]. W.H. Heinrich identified technical factors including equipment failures and design or manufacturing defects contributing to accidents [14]. Traditional accident risk models typically analyze single risk factors, which can result in biased estimates [15]. Williams and Tefft, for instance, observed increased accident risks among teenage drivers linked to factors such as speeding, drinking, nighttime driving, and failure to use seat belts, particularly with multiple teenage passengers [16]. Johnson et al. noted that marijuana use alone did not significantly affect accident risk, but age significantly influenced results when introduced as a variable [17]. Recent studies have shifted towards multiple risk factors. Wang et al. explored relationships between accident frequency and variables such as population, road density, and trip frequency, utilizing a Bayesian conditional autoregressive negative binomial model [18]. Lee et al. linked increased accident severity with compact cars, young or female drivers, heavy rainfall, and roads with poor drainage [19]. Various models have been proposed, including hierarchical ordered logit models [20], mixed multinomial logit methods [21], logistic regression [15,22], Bayesian methods [23,24], and artificial intelligence techniques [25]. However, these traditional methods often inadequately differentiate risk factor impacts, resulting in inaccurate conclusions [26,27]. Although traditional methods have laid the foundation for traffic accident analysis and responsibility attribution, the framework combining deep learning and Shapley value analysis has potential in this field. This framework can provide more detailed explanations for complex accident scenarios, significantly improving the accuracy and interpretability of responsibility attribution.

### 2.2. Factors impacting violation

This section explores the multi-dimensional factors influencing driver violations. Driver characteristics, including age and gender, play a significant role in driving behavior. Studies show young male drivers exhibit more dangerous habits, while females tend to be more cautious [26,27]. This may be due to physiological and psychological differences, with males seeking excitement and females prioritizing safety [28,29]. As drivers age, experience increases, but older drivers tend to suffer more severe injuries [30,31]. Research by Kim et al. and Cullen et al. supports these findings [32,33]. However, some studies, like Leitgeb et al., suggest gender has less impact on crash-related mortality after controlling for age [34]. Psychological traits also affect driving. Impulsive, aggressive individuals with a high-risk preference are more likely to violate traffic rules [35,36]. Angry driving, identified as a predictor of aberrant behavior, increases accident risk through actions like speeding and honking [37]. Emotional states, such as anxiety or the desire for excitement, further influence driving behavior across age groups [38]. Tanglai et al. found that factors like higher education and anger are linked to dangerous driving [39]. Environmental conditions, such as weather and road conditions, are critical. Bad weather, like fog, increases accident severity due to reduced visibility [40]. Road conditions also affect accident frequency and severity, with intersections being particularly hazardous [41]. Daylight reduces accident severity, as visibility is lower at night, highlighting the importance of proper lighting [42]. Other factors, such as road infrastructure and traffic signals, influence driver behavior [43–45].

In conclusion, the factors influencing driver violations are diverse, combining individual traits with environmental conditions. Understanding these is essential for developing effective safety interventions and improving road safety [18].

### 2.3. Methods of determining accident responsibility

Accident responsibility determination significantly impacts involved parties’ rights and societal justice, evolving with technological advancements.

The division of responsibility typically depends on several factors [3,5]: driver violations (e.g., distracted driving, fatigue, drunk driving, speeding, signal violations), environmental conditions (weather, road surface, visibility, traffic volume), vehicle conditions (mechanical failures, brake issues, lighting malfunctions), third-party factors (improper actions by other vehicles or pedestrians, animals), and emergency response measures (e.g., sudden braking, sharp turning).

Recent research has advanced intelligent methods for responsibility recognition. Liu et al. developed a system using case-based reasoning and D-S evidence theory to determine accident responsibility through similarity analysis and probability calculations [46]. Li et al. created a knowledge-based expert system simulating enforcement procedures to infer responsibilities and generate accident documents [47]. Tao et al. applied artificial intelligence in developing an intelligent decision support system for traffic accident responsibility recognition [48]. Although these studies have made significant progress in traffic accident responsibility attribution, they still have some limitations. Their systems’ knowledge bases are limited, which may lead to biases in responsibility recognition, and they may face challenges when handling complex accidents with multiple interrelated factors. Based on this, to better identify responsibility allocation and improve the explanation of responsibility attribution, this paper employs the AMFormer model to investigate intelligent responsibility recognition in traffic accidents. In the responsibility recognition process, deep learning methods are combined with SHAP explainability to analyze cases in the case database.

## 3. Methods

### 3.1. Overview

**Fig 1:**
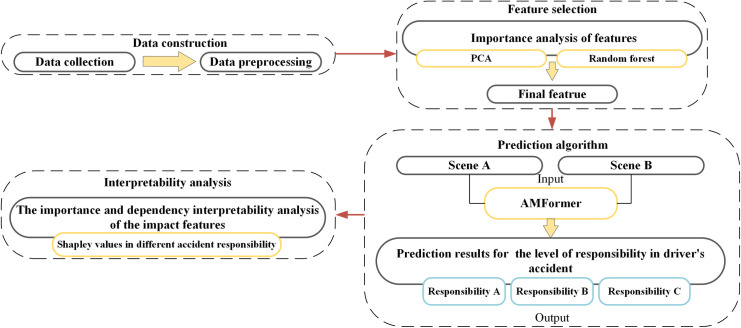
An analytic framework using deep learning for prediction of the level of responsibility in traffic accidents based on contributing factors.

In this paper, we propose an analytical framework based on the AMFormer deep learning model to predict accident responsibility levels according to driver-related factors influencing violations. As shown in Fig 1, we first collected the traffic accident data set in Chongqing, followed by data preprocessing steps, such as handling missing values and outliers. Second, we employed Principal Component Analysis (PCA) for dimensionality reduction, retaining principal components that capture 95% of the variance to reduce redundant features. Subsequently, a Random Forest model was used to rank the remaining features relevant to accident responsibility, and low-relevance factors were removed. Third, based on the selected highly correlated features, we applied the AMFormer-based deep learning model to predict the responsibility levels, exploring differences in responsibility attribution across scenarios. Finally, using Shapley values, we performed multi-class interpretability analysis across different accident responsibility levels to study the influential factors for full responsibility, no responsibility, primary, secondary, and equal fault categories.

### 3.2. Data construction

With the advancement and maturity of monitoring systems and intelligent transportation systems, data available for traffic accident attribution is increasing rapidly. However, existing public datasets such as NGSIM [49] often lack sufficient sample sizes and feature diversity, which can lead to challenges in model convergence and even overfitting, ultimately falling short of reflecting the current reality of driving accidents. To address this, this study utilized the Chongqing traffic accident dataset, which includes data disclosed by relevant public security departments in Chongqing, China from January 2017 to November 2020. We believe that this three-year period provides a robust foundation for training the model, as it incorporates both regular and exceptional events, while still being recent enough to reflect current traffic conditions and relevant legal frameworks.

**Fig 2:**
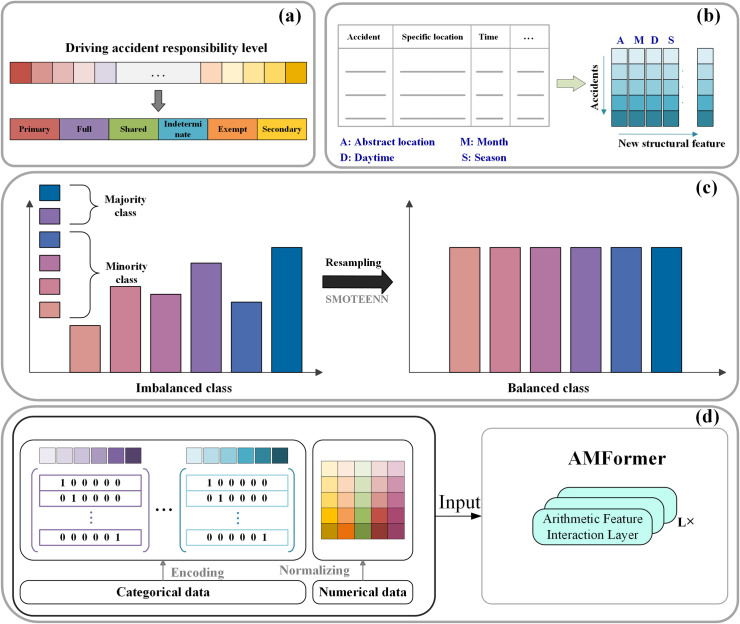
Data construction. (a) preparation of class labels (b) construction of new features (c) label resampling (d) Feature distinction.

Data preprocessing is essential to extract valuable information from the collected data. As shown in Fig 2, we began by performing data cleaning through summary statistics, deleting records with missing values, and replacing outliers with mean values. We also refined certain low-relevance features to enhance feature effectiveness. For example, while accident locations in the initial dataset were sometimes specific, road numbers and names were broader; therefore, we focused on segment types and extracted location keywords for classification to improve feature relevance. Additionally, we addressed label imbalance by applying the Synthetic Minority Over-sampling Technique – Edited Nearest Neighbors (SMOTE-ENN) technique, a method for imbalanced classification that combines Synthetic Minority Over-sampling Technique (SMOTE) with Edited Nearest Neighbors (ENN). SMOTE first performs oversampling, and ENN then undersampling to remove potential noise and duplicate samples, enhancing data balance and improving the generalization ability of the prediction model. Finally, we differentiated between categorical and continuous features in the dataset, using normalization techniques for continuous features and LabelEncoder for effective numerical encoding of categorical features.

### 3.3. The prediction algorithm

The analysis of structured tabular data is a critical area in machine learning. The dataset in this study contains both numerical and categorical features. While efficient feature engineering was applied initially, data quality issues, such as noise, persist. Moreover, the dataset lacks inductive biases like temporal and local structures, presenting challenges. Traditional tree-based ensemble models like XGBoost, LightGBM, and CatBoost remain popular due to their robustness in handling data quality issues but depend heavily on feature engineering.

**Fig 3:**
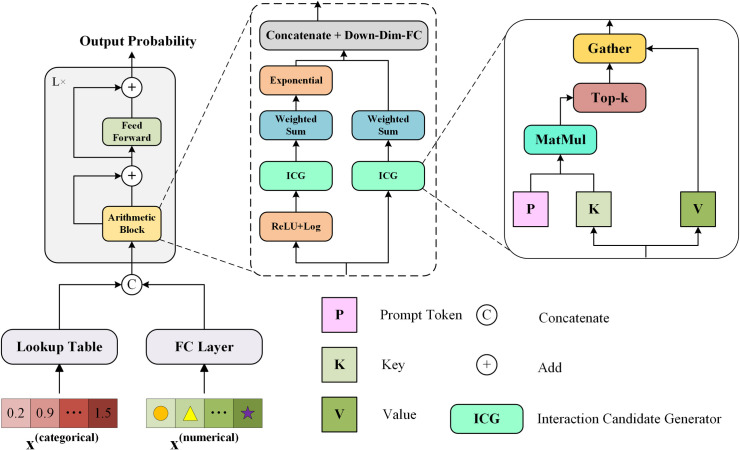
The overview of AMFormer.

The AMFormer algorithm in this study addresses the need for effective inductive biases in deep models for tabular data. It emphasizes arithmetic feature interactions and integrates this concept into the Transformer architecture. AMFormer, inspired by the classic Transformer, introduces an Arithmetic Block to enhance interaction with arithmetic features. As shown in Fig 3, AMFormer converts raw features into embeddings: a 1-in d-out linear layer for numerical features and a d-dimensional embedding lookup table for categorical features. These embeddings pass through *L* sequential layers, where each layer’s arithmetic module applies additive and multiplicative attention mechanisms to enable arithmetic feature interactions. The final output is generated via a classification or regression head based on enriched embeddings.

The arithmetic module’s key components include parallel attention mechanisms and prompt tokens. AMFormer configures parallel attention flows to extract meaningful additive or multiplicative interactions, which are fused through a downsampling linear layer. This setup enables AMFormer to capture arithmetic operations between features effectively. Formally, let X denote the number of input features at a specific layer l∈{1,…,L}, and let $M\;\in \;{\mathbb{R}}^{X\times d}$ represent the corresponding feature embedding matrix. Due to the inherently additive nature of the traditional attention mechanism, logarithmic scaling, as described in Equation (1), is applied in the multiplicative attention flow. This is then combined with exponential operations in the logarithmic space to learn multiplicative feature interactions.


$$\begin{array}{c} M_{\log}=\log\left(ReLU(M)+\epsilon \right) \end{array}$$ (1)

where ∊ prevents log0. For the additive flow, the query $Q=MW^{Q}$, key $K=MW^{K}$, and value $V=MW^{V}$ are first generated through the trainable parameters $W^{Q/K/V}\in {\mathbb{R}}^{d\times d}$). The output of the additive attention flow is computed as follows:


$$\begin{array}{c} O^{A}=\text{softmax}\left(\frac{QK^{T}}{\sqrt{d}}\right)V\in {\mathbb{R}}^{X\times d} \end{array}$$ (2)

The output establishes dense connections between every pair of features. However, feature interactions in tabular data are typically sparse. Therefore, the algorithm retains the highest number of entries in each row of $QK^{T}$and mask the remaining entries with negative values. As a result, each feature only interacts with k other features, ensuring sparse interactions. The improved output is as follows:


$$\begin{array}{c} O^{A}=\text{softmax}\left(\frac{top_{k\left(QK^{T}\right)}}{\sqrt{d}}\right)V\in {\mathbb{R}}^{X\times d}\; \end{array}$$ (3)

Similarly, for the multiplicative attention flow, the output $O^{M}\in {\mathbb{R}}^{X\times d}$ is obtained by applying logarithmic scaling, exponential operations, and another set of trainable parameters. Finally, the outputs of the additive flow $O^{A}$ and the multiplicative flow $O^{M}$ are concatenated vertically and fused through a fully connected layer to integrate the interaction candidates, as shown in the following equation:


$$\begin{array}{c} O=FC(VConcat(\left[O^{A},O^{M}\right]{)}^{T}{)}^{T} \end{array}$$ (4)

The computational complexity of the AMFormer model is primarily driven by the attention mechanisms and prompt optimization. For each layer, the complexity of the additive and multiplicative attention flows is $O(N^{2}\cdot d)$, where N is the number of features and d is the embedding dimension. However, with the use of top-k selection, the effective complexity can be reduced to O(k·N·d), where k is a small constant. Additionally, the introduction of prompt tokens optimizes the model’s complexity, reducing it to O(P·N·d), where P represents the number of prompt tokens, which is typically much smaller than N. Therefore, the overall complexity of AMFormer per layer can be expressed as O(L·P·N·d) with prompt optimization, where L is the number of layers. This optimization helps AMFormer efficiently handle large feature sets by significantly reducing computational and memory overhead.

AMFormer is designed to capture and process a variety of factors that influence traffic accident liability. Key driver-related factors such as driving behavior, experience, and state (e.g., alcohol consumption, fatigue) are input into the model, alongside environmental factors like weather, road conditions, and traffic density. These factors are handled through the model’s multi-label classification framework, where the interactions between these variables are learned through the network’s hidden layers. Through spatiotemporal feature modeling, AMFormer effectively mimics human expert judgment, which often takes into account the relative weight of each factor in determining responsibility. For instance, expert evaluators would likely assign a higher degree of responsibility to a driver involved in an accident due to speeding or drunk driving, while accounting for mitigating factors such as poor road conditions or bad weather. In AMFormer, these weights are learned from historical accident data, allowing the model to weigh the impact of different factors based on real-world accident scenarios.

### 3.4. The interpretability analysis

After using AMFormer for prediction, we intuitively apply Shapley values to flexibly select different features, thereby explaining the complex nonlinear behaviors of features in the process of an incident. Based on game theory, Shapley values combine optimal credit allocation methods with local explanations to provide effective interpretations of attribute parameters. Therefore, we use them to explain the outputs of the AMFormer model. This method is commonly used to describe the relative importance of *N* features or to assess the impact of feature behaviors. In recent years, the SHAP (Shapley Additive Explanations) method, based on a unified framework, has been proposed. Its core idea is to compute the independent contributions of features to model predictions using Shapley values from cooperative game theory, enabling the quantitative comparison of feature contributions and providing a universal explanation across various models.

Let the feature set be $N=\{x_{1},x_{2},\ldots ,x_{n}\}$. The goal is to compute the marginal contribution of a specific feature $x_{i\;}$to the model’s prediction, i.e., the SHAP value $\phi_{i}$, defined as follows:


$$\phi_{i}=\sum_{S\subseteq N\setminus \{i\}}\frac{|S|!\left(n-|S|-1\right)!}{n!}\left[f\left(S\cup \left\{i\right\}\right)-f(S)\right]$$ (5)

where S⊆N∖{i} represents all possible subsets of the feature set *N* excluding the feature $x_{i\;}$, f(S) denotes the model’s prediction given the feature subset *S*, and f(S∪{i})−f(S) calculates the marginal contribution of feature $x_{i\;}$ to the prediction when added to subset *S*.

SHAP provides both global and local interpretability. On the global level, SHAP values reflect the contribution trends of each factor to the AMFormer model’s predictions in the context of driving accidents, helping us understand the importance of different features. On the local level, SHAP values reveal the feature contributions for specific samples, explaining the prediction logic of AMFormer under specific input conditions. We will discuss this in detail in Section 4.

## 4. Experiment and results

### 4.1. Data description and analysis

To evaluate the effectiveness and applicability of the proposed analytical framework, this study utilizes a dataset of traffic accidents in the Chongqing area. The dataset, provided by transportation authorities, covers the period from January 1, 2017, to December 31, 2019, and was fully anonymized prior to being shared with the research team. The authors did not have access to any information that could identify individual participants during or after data collection, ensuring data privacy and compliance with ethical standards.

The dataset includes various factors related to the circumstances surrounding each accident. To enhance the analysis, we ultimately selected 50 additional influencing factors for dimensionality reduction to assess the proposed framework. Tables 1 and 2 respectively provide statistical analysis of key categorical and numerical features in the data. After organizing the dataset, we performed thorough data cleaning on the traffic accident records, including the removal of missing values and the handling of outliers.

**Table 1:** Partial descriptive statistics for categorical features.

Feature	Category	Proportion (%)
Personnel characteristics		
Gender	Male	77.90
	Female	22.10
Pedestrian status	Cross the crosswalk	15.26
	Cross the road	42.72
	Stop on the road	5.36
	Walking on a motorway	7.67
	Go over the containment facility	7.61
	Normal passage	21.38
Pedestrian speed	Running	12.15
	Normal walking	62.87
	Walking slowly	9.21
	Walking quickly	7.21
	Static	8.56
Vehicle characteristics		
Vehicle type	minibus	30.36
	Medium bus	0.89
	Large bus	1.14
	motorcycle	20.61
	minivan	3.71
	Light truck	0.03
	Medium truck	0.73
	heavy truck	4.88
	tractor	0.48
	semi-trailer	0.03
	Full trailer	0.01
	Special operation vehicle	0.64
	Other	36.49
Mode of transport	Drive a car	41.49
	Drive a motorcycle	19.99
	Drive non-motor vehicles	4.86
	Drive a tractor	1.47
	Drive a farm transporter	1.08
	Driving other motor vehicles	31.11
Accident location	Normal road	51.00
	Rural	5.62
	Bridge	5.86
	Notional road	6.83
	County road	0.67
	Cross section	1.84
	Provincial road	16.93
	Residential area	2.23
	Hub	1.80
	Near public facilities	2.70
	Industrial park	1.14
	Highway	0.61
	Tunnel	0.80
	Express road	0.47
	Circle line	0.04
	Other	1.47
Environmental characteristics		
Weather	Cloudy	29.80
	Sunny	51.80
	Rain	18.12
	Fog	0.19
	Snow	0.04
	Other	0.04
Visibility	50 meters below	9.81
	50-100 meters	23.47
	100-200 meters	21.71
	200 meters above	45.01
Lighting conditions	Dawn	3.32
	Daytime	57.00
	have a street lighting at night	26.84
	without a street lighting at night	10.03
	twilight	2.71
	tunnel without lighting during the day	0.06
	tunnel has lighting during the day	0.04
Terrain	Hilly	65.89
	Mountainous	27.25
	Plain	6.86

**Table 2:** Descriptive statistics for numerical features.

Feature	Mean	SD	Min	Max	25% quantile	50% quantile	75% quantile
Total deaths	0.255	0.506	0.0	8.0	0.0	0.0	0.0
Injuries	1.324	1.234	0.0	23.0	1.0	1.0	2.0
Kilometers	828.061	834.448	0.0	9048.0	85.0	1001.0	1005.0
Meters	198.935	328.591	0.0	9999.0	10.0	100.0	300.0
Age	44.387	17.159	0.0	80.0	31.0	45.0	54.0
Driving years	9.346	6.109	1.0	46.0	5.0	8.0	13.0

When examining temporal characteristics, we split the original accident occurrence time into two fields: date and hour. The hour field helps account for periods such as the morning and evening rush hours, afternoon drowsiness, and late-night fatigue. The date field considers seasonality, dividing the data by time of day and seasonal features. Subsequently, to capture more relevant features, we conducted descriptive statistics and machine learning feature analysis on the two-year dataset (2017–2019). After cleaning, the original dataset includes 40,120 accident records involving a total of 46,286 vehicles and 8,267 individuals, containing multiple variables that may influence accident responsibility attribution. Accident responsibility is categorized into six types: Primary Responsibility, Full Responsibility, Shared Responsibility, Indeterminate Responsibility, No Fault or Exempt from Responsibility, and Secondary Responsibility. The distribution of these categories is as follows: Primary Responsibility, 5,841 cases (14.6%); Full Responsibility, 9,040 cases (22.5%); Shared Responsibility, 4,493 cases (11.2%); Indeterminate Responsibility, 364 cases (0.9%); No Responsibility, 14,369 cases (35.8%); and Secondary Responsibility, 6,012 cases (15.0%).

### 4.2. Data process and experiment setup

#### 4.2.1. Data preprocessing.

In this data processing, we applied rigorous data cleaning and feature engineering to enhance model prediction performance. First, during data cleaning, we systematically addressed duplicates, missing values, and outliers. Duplicate data can bias the model, so we removed identical samples using a deduplication algorithm to ensure data independence and diversity. For handling missing values, we applied different strategies based on the proportion of missing data and feature importance. Columns with high proportions of missing values were removed to avoid potential biases, while key columns with lower missing rates were filled using mean imputation to preserve data quantity without significantly altering distribution characteristics. Outliers were detected using the z-score method, which identifies anomalies based on mean and standard deviation. By calculating each sample’s z-score and identifying those exceeding a set threshold, we flagged extreme data points. These outliers were then replaced with the feature mean to mitigate their influence on model training and prevent bias. Next, we conducted an in-depth analysis and processing of the dataset’s variables to enhance feature validity and relevance. In the initial dataset, accident location information included specific segments as well as broader road names or numbers. We refined this feature by categorizing accident locations based on key segment types, improving the association between accident location and prediction targets. We also examined other potentially low-relevance features, converting or re-encoding them where necessary to construct a more concise and effective feature set.

To address data imbalance, we employed the SMOTE-ENN method, combining oversampling and undersampling techniques. Specifically, SMOTE generated synthetic samples for minority classes, such as shared responsibility, balancing the dataset, and reducing model bias toward majority classes. ENN further refined the synthetic dataset by removing noise points and low-quality samples, enhancing overall dataset quality and balance. This approach effectively improved model performance on minority classes and enhanced its generalization ability.

Finally, in the data preprocessing phase, we process the continuous and categorical features respectively. For continuous features, we applied normalization techniques (such as Min-Max Scaling or Standard Scaling) to reduce differences in scale and unit inconsistencies, accelerating model convergence and improving accuracy. For categorical features, we used LabelEncoder to numerically encode categorical labels, converting them into a form suitable for machine learning models, thus enhancing the model’s ability to interpret categorical features. These steps, including preprocessing, feature extraction, imbalance handling, and encoding, ensured the high quality and consistency of the dataset, providing a solid foundation for subsequent model training.

#### 4.2.2. Experiment setup.

To ensure consistency in the experimental environment, the model training and testing were conducted on the same platform. The hardware configuration includes a 16-core AMD EPYC 7302 @2.4GHz CPU and an NVIDIA GeForce RTX 3090 with 24GB of memory. The operating system used is Ubuntu 18.04, with PyTorch 1.11.0 as the deep learning framework, PyCharm as the development platform, CUDA 12.1, and Python 3.8.

The training configuration parameters for each model are as follows: The batch size is set to 128, with 100 epochs. Warmup pretraining is conducted for three epochs with an initial learning rate of 0.01, followed by a cosine annealing schedule to dynamically adjust the learning rate, ultimately reaching 0.1. The momentum factor beta1 is set to 0.937, and the weight decay rate is 0.0005.

### 4.3. Feature selection and correlation analysis

#### 4.3.1. Feature selection using machine learning approaches.

To optimize the dataset for accident responsibility analysis and improve the model’s efficiency and interpretability, this study employed machine learning algorithms for feature selection. The process was divided into two main steps: dimensionality reduction using PCA and feature ranking and filtering based on the Random Forest algorithm.

First, PCA was applied to reduce the dimensionality of the data, addressing issues of redundancy and multicollinearity. Specifically, PCA transforms the original features into a set of new, mutually orthogonal principal components, which are ranked according to their contribution to the total variance in the data. In this study, we selected principal components that explained 95% of the cumulative variance. This approach significantly reduced the number of features while retaining the most critical information, ensuring data simplification without losing key insights. After dimensionality reduction, the Random Forest algorithm was used to further assess the importance of the remaining features. As a robust ensemble learning algorithm, Random Forest quantifies feature importance by measuring their contribution to splitting nodes across all decision trees. Additionally, it evaluates feature significance by observing changes in model prediction error before and after shuffling each feature. Based on the importance scores from Random Forest, ten low-contribution features were removed, including “Accident Number,” “Administrative Division,” and “Accident Cause Identification.” These features were either redundant (e.g., identifier-type features) or weakly correlated with accident responsibility prediction.

This systematic feature selection process not only significantly reduced data dimensionality and model complexity but also enhanced computational efficiency and predictive performance. Ultimately, we retained core features closely related to accident responsibility, improving the analysis’s interpretability and scientific rigor, and providing a reliable data foundation for subsequent modeling.

#### 4.3.2. The correlation of the feature.

**Fig 4:**
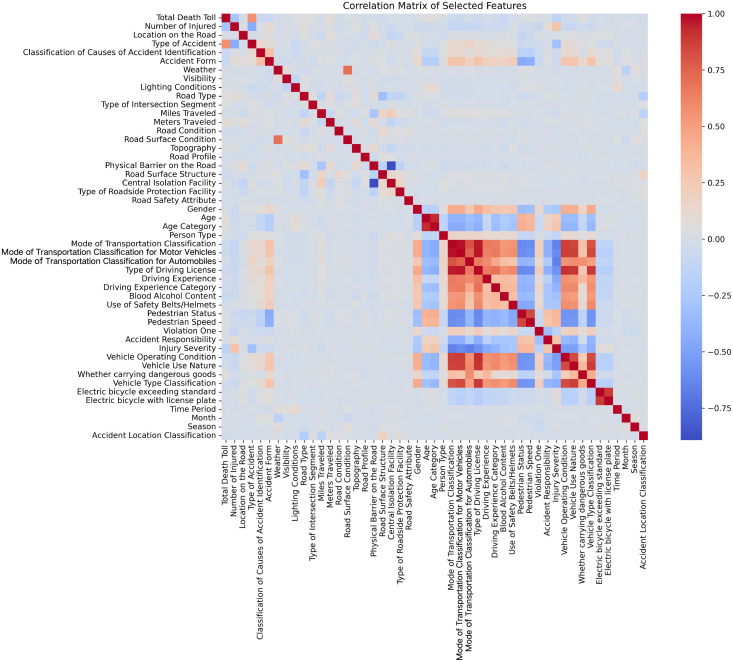
Correlations among predictors (Blue grids represent a strong positive correlation and red grids represent a strong negative correlation).

In the research process, analyzing the correlations between features is a crucial aspect of data processing. Fig 4 illustrates the correlation matrix of the primary features in the dataset, where the Pearson correlation coefficients between each pair of features are visualized using color gradients and polarity. Specifically, red represents positive correlations, blue represents negative correlations, and darker colors indicate stronger correlations. Observing the correlation matrix reveals a certain degree of multicollinearity in the data.

It is evident from the matrix that certain features are strongly correlated. For example, “Driving Classification” shows a significant positive correlation with features related to “Motorized Vehicle Classification in Transportation Modes.” Additionally, variables related to road conditions (e.g., “Road Safety Attributes” and “Pavement Conditions”) and environmental factors (e.g., “Weather” and “Lighting Conditions”) also exhibit strong correlations. For example, under adverse weather conditions (such as rainy or foggy weather), the friction coefficient of the road surface may decrease, leading to an increase in the vehicle’s braking distance. The interaction between these road conditions and environmental factors (such as low visibility due to weather) significantly raises the probability of an accident occurring, especially when driving at high speeds. Similarly, at night, poor road conditions or roads with insufficient lighting can cause delays in the driver’s response to obstacles, thereby increasing the risk of an accident. Therefore, the interaction between road conditions and environmental factors can significantly impact both the occurrence of traffic accidents and the determination of responsibility. Such high correlations suggest that different features may share similar information, making it challenging to independently evaluate the effects of certain variables in the model. Multicollinearity is a common issue in statistical modeling and predictive analysis. When multiple features are highly correlated, the model may perform well on training data but poorly on test data, leading to overfitting or underfitting. Furthermore, when features share similar information, it becomes difficult to distinguish each feature’s specific contribution to the predictive target, potentially compromising the model’s interpretability. Although removing highly correlated features is one way to mitigate multicollinearity, this approach may result in the loss of important information. For instance, variables related to transportation modes, driving behavior, and road safety may be highly correlated but represent distinct dimensions; their removal might obscure key mechanisms. Thus, relying solely on feature removal is not a comprehensive solution.

To balance feature retention and model robustness, we adopted a modeling technique better suited for handling multicollinearity. AMFormer, an advanced deep learning model designed for spatiotemporal feature modeling, addresses this challenge effectively. Unlike traditional linear models, its nonlinear nature enables it to extract meaningful patterns from highly correlated features. By leveraging a multi-head attention mechanism to dynamically balance the relative importance of features, AMFormer mitigates the negative impact of feature correlations on modeling.

In this paper, we ended up keeping 50 features and incorporated them into the AMFormer model. This approach not only preserves potential information to the greatest extent but also utilizes AMFormer’s modeling capabilities to address the effects of multicollinearity on model stability and predictive performance. Moreover, AMFormer’s attention mechanism supports feature importance analysis, providing a solid foundation for subsequent interpretability studies.

### 4.4. Model evaluation metrics

In this study, we use Accuracy (A), Precision (P), Recall (R), and F1-score to evaluate model performance.

Accuracy refers to the proportion of correctly predicted samples out of the total samples. It is calculated as:


$$\text{Accuracy}=\frac{\text{TP+TN}}{\text{TP + TN+FP+FN}}$$ (6)

Precision represents the proportion of actual positives among all samples predicted as positive, primarily measuring the model’s accuracy in positive predictions. It is calculated as:


$$\text{Precision}=\frac{\text{TP}}{\text{TP + FP}}\;$$ (7)

The recall represents the proportion of correctly predicted positive samples among all actual positive samples, primarily measuring the model’s ability to identify positive samples. It is calculated as:


$$Recall=\frac{TP}{TP+FN}$$ (8)

F1-score is the harmonic mean of Precision and Recall, providing a balanced measure of the model’s accuracy and coverage. It is commonly used as an evaluation metric when both Precision and Recall are important. The formula is as follows:


$$F1-socre=\frac{2\times Precision\times Recall}{Precision+Recall}\;$$ (9)

where TP (True Positive) represents the number of samples that are positive and predicted as positive, TN (True Negative) represents the number of negative samples and predicted as negative, FP (False Positive) represents the number of samples that are negative but predicted as positive, and FN (False Negative) represents the number of samples that are positive but predicted as negative.

### 4.5. Hyperparameter tuning

Table 3 summarizes the hyperparameters optimized for machine learning and deep learning models. The hyperparameter selection was guided by three principles: (1) improving predictive performance to enhance the model’s representational capacity; (2) preventing overfitting by leveraging regularization and appropriate hyperparameter settings to strengthen the model’s generalization ability; and (3) achieving a balance between predictive performance and computational cost.

**Table 3:** Hyperparameters of prediction model.

No.	Prediction algorithm	Hyperparameters
1	AMFormer	Embedding Dimension: 192, Number of Attention Heads: 8, Depth:5, Feedforward Layer Dimension: 256, Atten_dropout: 0.1,
2	Random Forest	Number of Trees (n_estimators): 100, Max Depth: None, Min Samples Split: 2, Min Samples Leaf: 1, Criterion: Gini
3	SVM	Kernel: RBF, Regularization Parameter (C): 1.0, Gamma: Scale, Tolerance: 1e-3
4	GBTD	Number of Trees (n_estimators): 200, Learning Rate: 0.1, Max Depth: 3, Min Child Weight: 1, Subsample: 0.8
5	CatBoost	Iterations: 500, Learning Rate: 0.03, Depth: 6, L2 Leaf Regularization: 3.0, Loss Function: CrossEntropy
6	FT-Transformer	Embedding Dimension: 64, Number of Attention Layers: 6, Dropout: 0.2
7	AutoInt	Embedding Dimension: 128, Number of Interaction Layers: 3, Dropout: 0.1
8	TabNet	Number of Decision Steps: 5, Feature Dimension: 64, Attention Dimension: 32, Gamma: 1.5

For the AMFormer deep learning model, we tuned several key hyperparameters, including the embedding dimension, which was set to 192 for a balance between performance and computational efficiency. The number of attention heads was chosen to be 8, as it provided the best feature interaction modeling, while the depth of the network was set to 5 to avoid overfitting while capturing complex patterns. The feedforward layer dimension was set to 256, which effectively handled the complexity of the dataset, and a dropout rate of 0.1 was applied to the attention layers to mitigate overfitting. For the Random Forest model, we optimized the number of trees (n_estimators) to 100, which gave a good trade-off between accuracy and computation time. The maximum tree depth was left unconstrained to allow the trees to fully capture the data’s complexities, while the minimum samples required to split an internal node (min_samples_split) were set to 2, and the minimum samples per leaf (min_samples_leaf) were set to 1. In the case of the Support Vector Machine (SVM), we adjusted key parameters such as the kernel type and regularization parameter (C), setting the C parameter to 1.0 to control overfitting and improve model generalization. For Gradient Boosted Decision Trees (GBDT) and CatBoost, we fine-tuned parameters like the number of trees, learning rate, and tree depth to ensure the model could capture both the linear and non-linear relationships within the data. Hyperparameter tuning was also performed for other deep learning models, such as FT-Transformer, AutoInt, and TabNet, where parameters like embedding dimensions, number of attention layers, and interaction layers were adjusted to optimize their feature extraction and learning capabilities. Each model’s parameters were carefully selected based on cross-validation and grid search, ensuring the final model configurations balanced predictive power and computational efficiency. To save computational resources, we used default values for parameters less sensitive to changes (e.g., optimization functions and activation functions) and focused on optimizing hyperparameters that had a significant impact on predictive performance. All models underwent meticulous hyperparameter tuning, and only the best-performing results were reported in subsequent analyses, providing a robust foundation for further research.

### 4.6. Evaluating performance comparison

To validate the effectiveness of the AMFormer model, this section conducts comparative experiments with several mainstream machine learning and deep learning models, including Random Forest, LGBM, GBDT, CatBoost, FT-Transformer, AutoInt, and TabNet. Evaluation metrics include Accuracy, Precision, Recall, and F1-score, with the experimental results summarized in Table 4.

**Table 4:** Performance comparison of different models.

Algorithm	Accuracy (%)	Precision (%)	Recall (%)	F1-score (%)
**AMFormer**	**93.46**	**93.42**	**93.25**	**93.33**
Random Forest	85.65	85.49	85.53	85.51
LGBM	84.93	84.25	85.49	84.87
GBTD	82.55	82.51	83.60	83.05
CatBoost	85.88	85.95	86.66	86.30
AutoInt	87.96	87.29	87.08	87.18
TabNet	88.03	87.99	88.01	88.00

The results demonstrate that AMFormer outperforms all other models across all evaluation metrics. Specifically, its Accuracy, Precision, Recall, and F1-score reached 93.46%, 93.42%, 93.25%, and 93.33%, respectively, showcasing superior adaptability to multi-class labels in accident responsibility prediction tasks. In contrast, traditional machine learning models, while robust in feature importance analysis and handling categorical data, struggle to capture the complex nonlinear relationships and fuzzy class boundaries within the dataset, resulting in metrics consistently below 90% and overall inferior performance. Deep learning models show advantages in handling high-dimensional features and capturing complex feature interactions. However, they fall short of accurately modeling arithmetic interactions between driver behavior, environmental conditions, and road characteristics. FT-Transformer, as a precursor to AMFormer, achieves slightly higher performance than other mainstream models, with an F1-score of 90.20%. Nevertheless, it still exhibits limitations in capturing intricate feature interactions.

The superior performance of AMFormer stems from its optimized arithmetic feature interaction module and multi-head attention mechanism. This design efficiently captures complex nonlinear relationships between numerical and categorical features in the dataset while leveraging the high-correlation variables selected through PCA and Random Forest. These enhancements significantly improve the model’s adaptability to responsibility classification, enabling it to surpass its competitors comprehensively.

### 4.7. Multi-Level Shapley analysis

**Fig 5:**
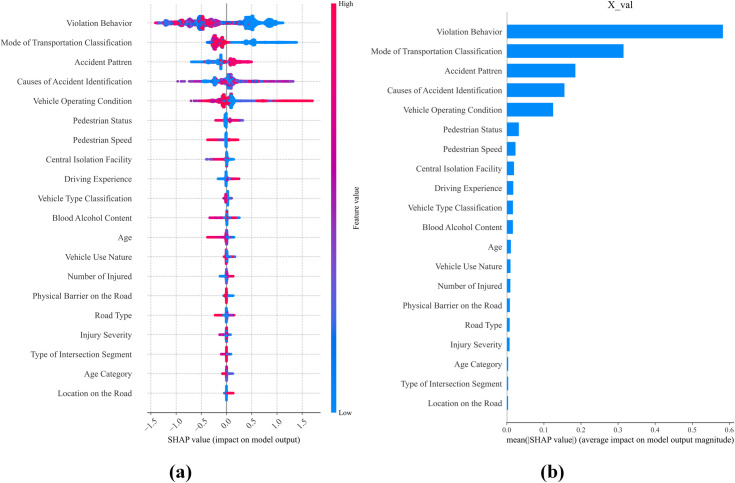
Global SHAP summary plot.

In this section, we conducted an interpretability analysis of the model using SHAP values. Fig 5 displays the average importance of each feature in accident responsibility classification, ranked from high to low based on their impact, highlighting the distribution and role of the top 20 key features. The analysis reveals that accident-related factors play a critical role in determining responsibility, with Violation Behavior being the most important feature among these factors. Its influence on responsibility prediction significantly outweighs that of other features. This finding underscores the core role of the severity of violation behavior in accident responsibility determination, emphasizing that the direct nature of violations dictates their dominant role in assigning responsibility. It also further stresses the importance of enhancing driver behavior regulation and education. Accident Pattern, the second most important feature within the accident factors, provides valuable support for model interpretability. By describing the specific form of the accident (e.g., collision, rollover, or skidding), it contributes to responsibility assignment in complex accident scenarios, particularly in multi-vehicle accidents or special situations.

Vehicle-related factors also play a crucial role in accident attribution. Mode of Transportation Classification, the most important feature within this factor, reflects the significant role of different modes of transportation (e.g., motor vehicles, non-motorized vehicles, pedestrians) in responsibility assignment. Especially in accidents involving motor vehicles, the type of transportation mode is a critical basis for responsibility determination, tightly linked to the legal framework and the actual conditions of the accident scenario. Vehicle Operating Condition, by indicating whether the vehicle is speeding or mechanically faulty, directly influences responsibility attribution.

Moreover, human risk factors play an essential role in traffic accident responsibility determination. Features like Pedestrian Status hold high importance as they reflect the direct causes of accidents and pedestrian behavior patterns (e.g., whether the pedestrian is jaywalking or crossing the road illegally). These features provide supplementary support for assigning responsibility in both single-vehicle and multi-vehicle accidents, effectively improving the accuracy of the model’s predictions. Additionally, Blood Alcohol Content remains a significant predictor of responsibility in cases involving drunk driving, where it can directly affect responsibility determination.

In contrast, road-related factors contribute relatively less to responsibility determination on average. However, features like Physical Barriers on the Road and Road Type still play a supplementary role in specific scenarios (e.g., roads lacking necessary infrastructure or complex traffic conditions at night). This suggests that while adverse road and environmental conditions can impact driving, accidents occurring in the absence of human factors tend to be less severe, resulting in lighter responsibility assignments. Measures that focus on reducing human risk factors would significantly alleviate the determination of accident responsibility.

Across four different dimensions of analysis, Violation Behavior emerges as the most critical factor influencing accident responsibility attribution across four analytical dimensions. High SHAP values highlight that severe violations like red-light running or speeding significantly increase responsibility levels, whereas minor infractions may have limited influence. Thus, violation behavior is crucial for accurately classifying responsibility. Among vehicle-related factors, Mode of Transportation Classification is highly influential. Motor vehicles, due to greater speed and mass, typically bear more responsibility compared to non-motorized vehicles or pedestrians. The SHAP value distribution underscores transportation mode’s importance in distinguishing responsibility, particularly in collisions involving different vehicle types, aligning closely with legal guidelines. Within human risk factors, Pedestrian Status significantly affects responsibility attribution. Non-compliant pedestrian behaviors (e.g., jaywalking or crossing highways) substantially elevate accident responsibility, while adherence to rules reduces pedestrian responsibility. This emphasizes human behavior analysis as essential for accurate responsibility assignments in complex scenarios. Regarding road-related factors, the Central Isolation Facility is paramount. SHAP analysis shows that roads lacking isolation facilities result in higher accident responsibility, particularly in high-traffic or highway conditions, due to increased lane departure and pedestrian crossing risks. These insights underline infrastructure’s critical role in reducing accident disputes and highlight the importance of implementing isolation facilities in high-risk areas.

All in all, Violation Behavior and Mode of Transportation Classification, as core features, dominate the model’s predictions, while features like Pedestrian Speed and Pedestrian Status enhance the responsibility classification framework with multidimensional supplementary information. This multilayered importance analysis not only reveals the hierarchical relationships between features but also provides a comprehensive theoretical for accident responsibility determination, improving the model’s transparency and interpretability.

**Fig 6:**
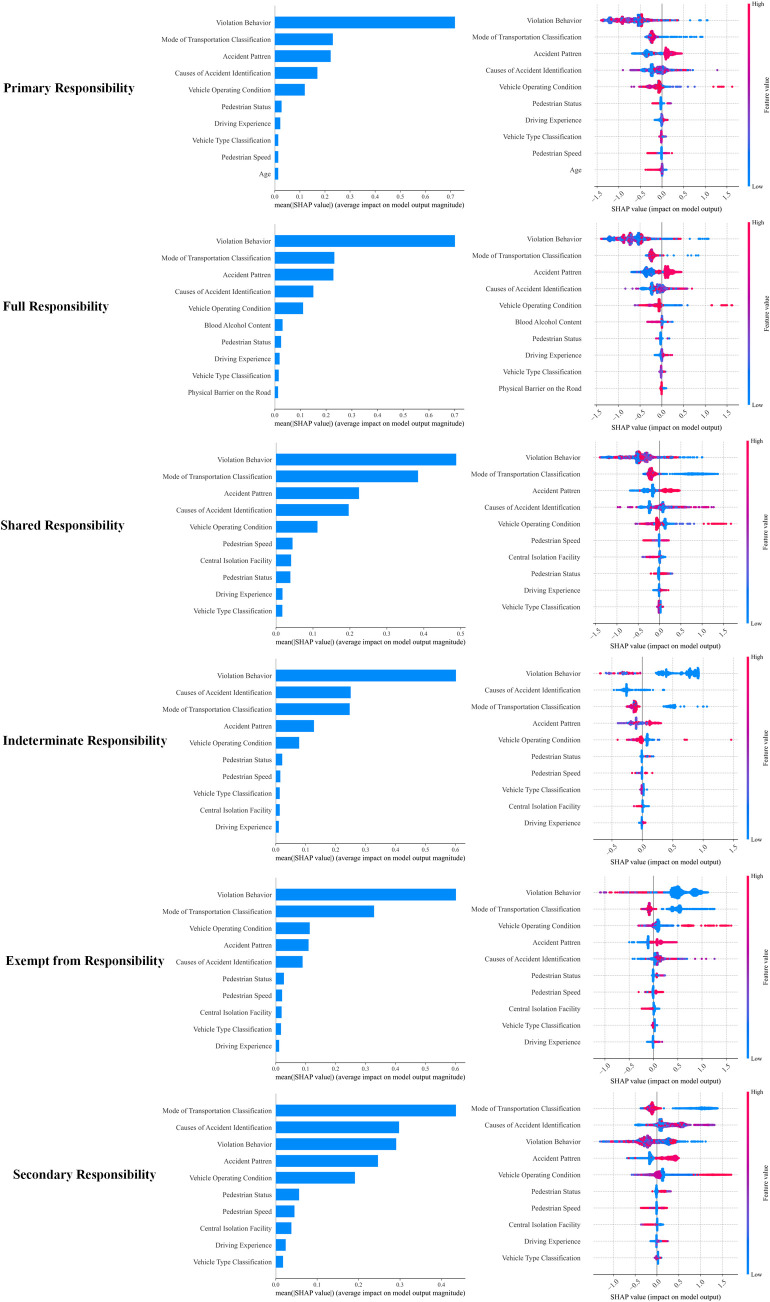
SHAP summary plot for different responsibility categories.

Fig 6 shows the average importance of each feature in accident responsibility classification under different responsibility categories. By analyzing different responsibility categories, we can gain deeper insights into the relative role and influence of each feature in the responsibility determination process.

In cases where no fault or exempt from responsibility is assigned, Vehicle Operating Condition becomes more important. This suggests that in accidents with no assigned responsibility, the vehicle’s condition—whether there is a malfunction or improper operation—plays a decisive role in responsibility assignment. The state of the vehicle often directly impacts the occurrence of the accident and the attribution of responsibility, especially in incidents caused by equipment issues where the vehicle’s condition becomes a key factor.

For accidents where full responsibility is assigned, Blood Alcohol Content significantly increases in importance. This feature is directly related to the driver’s behavior, particularly in accidents caused by drunk driving, where blood alcohol content becomes a crucial basis for assigning full responsibility. The impact of alcohol on the driver’s reaction time and judgment makes this factor especially prominent in full responsibility assignments. It is particularly relevant in specific accident scenarios, such as late-night or holiday periods when drunk driving accidents are more likely to result in full responsibility.

When responsibility is classified as indeterminate responsibility, Causes of Accident Identification become particularly important. In this situation, a detailed classification of the accident causes provides more information for responsibility determination, especially when evidence at the accident scene is insufficient or witness statements are contradictory. An in-depth analysis of the accident causes can help identify whether assignable factors exist, thus influencing the final responsibility classification.

In cases of primary responsibility, Human Factors, particularly Driving Experience, play a more significant role. This suggests that the driver’s behavior and experience are core elements in determining primary responsibility. Experienced drivers typically respond more effectively to emergencies, while novice drivers may struggle with timely reactions or judgment errors, leading to accidents and an increased responsibility assignment. Therefore, driving experience becomes a key criterion in determining primary responsibility.

For secondary responsibility, the Mode of Transportation Classification is the most critical feature. This indicates that, in cases of secondary responsibility, the type of transportation mode—such as motor vehicles, non-motorized vehicles, or pedestrians—has a significant impact. For example, in accidents involving motor vehicles and non-motorized vehicles, motor vehicles are often considered the primary responsible party due to their speed and mass, while non-motorized vehicles may be assigned secondary responsibility. The diversity of transportation modes also complicates the responsibility assignment process, with the mode of transportation playing a crucial role.

In cases of shared responsibility, Road Type and Weather Conditions become more prominent. This suggests that, in shared responsibility scenarios, the road’s condition (e.g., whether there are surface damages or traffic signs) and weather conditions (e.g., rain, snow, fog) play a significant role in responsibility allocation. Adverse weather and complex road conditions may lead to driver misjudgment or delayed reactions, thereby influencing both the occurrence of the accident and the attribution of responsibility.

**Fig 7:**
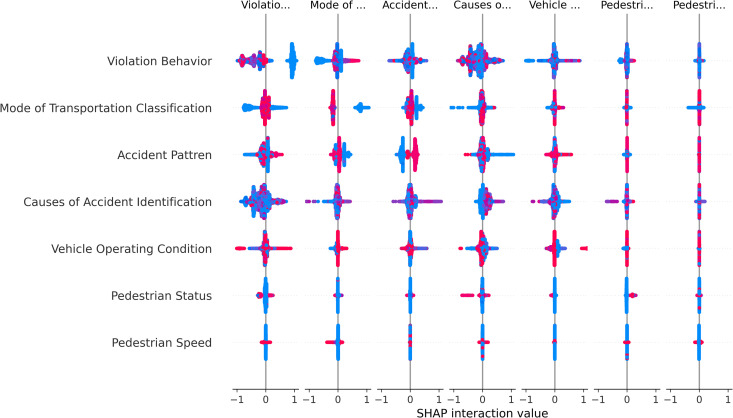
Interaction summary plot.

Fig 7 presents a visualization of feature interaction effects in the model based on the SHAP interaction analysis method. It reveals the contribution of feature interactions to the model’s prediction output and their interdependencies, providing a deeper quantitative analysis of the model’s interpretability. This analysis effectively identifies how the collaborative effects between features shape the decision-making process in complex predictive tasks.

The features are ranked according to their overall importance in the model’s predictions, with the most important features at the top. Violation Behavior is the most significant feature, and its interactions with other features play a decisive role in accident responsibility prediction. This suggests that, in addition to the independent influence of this feature on the model’s results, its combined effect with other features significantly enhances the model’s predictive accuracy and robustness when handling complex scenarios. For example, features such as Accident Pattern and Vehicle Operating Condition are key components of Violation Behavior, and together they exhibit strong synergistic effects when determining accident responsibility. The interaction between Mode of Transportation Classification and Accident Pattern is also highly important, indicating that the interaction between transportation mode and accident type is crucial in the model’s judgment of responsibility. These features interact to reveal how different modes of transportation and accident types jointly affect responsibility assignment. For instance, in fatal accidents involving motor vehicles, the responsibility is generally assigned in full to the driver. Additionally, in complex scenarios with multiple contributing factors, this interaction illustrates the model’s adaptability to multidimensional data.

Further analysis reveals that the interaction between Causes of Accident Identification and Pedestrian Status is highly concentrated, indicating significant collaborative effects in predicting responsibility for certain accident categories. This finding underscores the importance of combining pedestrian behavior and the cause of the accident in predicting responsibility. For example, when pedestrians cross road isolation barriers, the cause of the accident is often classified as “pedestrian crossing road isolation facility,” and in most cases, the pedestrian is assigned primary responsibility in determining accident responsibility. On the other hand, the interaction between Vehicle Operating Condition and Pedestrian Speed shows more dispersion, suggesting that these two features have considerable heterogeneity and do not interact directly. However, when a pedestrian is involved in a traffic accident, Pedestrian Speed becomes a key factor in determining the Violation Behavior and Causes of Accident Identification for both parties, ultimately influencing the assignment of responsibility.

The interpretability analysis performed using SHAP provides valuable insight into how AMFormer processes driver and environmental factors. The analysis reveals that, similar to human expert evaluators, AMFormer gives significant weight to driver behavior, particularly violations such as speeding or drunk driving, as well as environmental factors such as road conditions and weather. For example, the model assigns a higher responsibility level to accidents that occur under adverse weather conditions or in areas with poor road infrastructure, paralleling the judgment of expert evaluators. These findings demonstrate how the model mimics expert human judgment, making its predictions more transparent and interpretable.

## 5. Discussion

Accident responsibility attribution significantly impacts traffic safety management, fairness, and compensation accuracy [49]. Traditional attribution methods rely on subjective human judgment, often leading to biases [4]. Deep learning offers improved automatic factor extraction and interpretability, enhancing accuracy and efficiency in responsibility attribution [7,8]. Addressing this gap, we propose an AMFormer-based deep learning framework that combines prediction and interpretable analysis, thereby increasing both accuracy and transparency [50–53].

One significant finding of this study is that AMFormer consistently outperforms traditional models (Random Forest, LGBM, GBDT) across evaluation metrics, demonstrating superior adaptability for multi-class responsibility prediction [54]. Shapley value analysis further highlights critical explanatory factors [55]. “Violation Behavior” emerged as the most influential factor, emphasizing its decisive role in responsibility determination and the need for strict driver regulation. “Mode of Transportation Classification” and “Accident Pattern” follow closely, highlighting vehicle type and accident context as essential elements [32,56,57]. Additionally, “Causes of Accident Identification” and “Pedestrian Status” significantly contribute to predictive accuracy, particularly in complex scenarios involving pedestrians and external factors [47,58]. The findings indicate that driver violations primarily influence responsibility attribution, whereas environmental factors become prominent when driver behavior is standard [59,60]. These insights validate the model’s effectiveness and offer practical guidance for traffic management decisions [61].

While SHAP provides valuable insights into the model’s behavior, it is not immune to biases introduced by the underlying data, especially if the dataset lacks sufficient diversity in certain scenarios. Additionally, SHAP is a post-hoc explanation method, meaning that it can only offer an interpretation of what the model has learned, but it does not directly explain how the model arrived at its decision in every case, particularly in more complex or unseen scenarios. Furthermore, as with any explainability tool, SHAP’s explanations are constrained by the model’s architecture and may not fully capture the intricacies of human judgment in legal and traffic safety contexts. Nonetheless, the feedback from the experts supports the validity of the SHAP explanations in this context, while recognizing that further refinement and validation with a wider range of domain experts would be beneficial for enhancing the model’s real-world applicability.

It is worth noting that the liability predictions made by the AMFormer model were cross-referenced with legal guidelines for traffic accidents in China. The model’s attribution of responsibility levels (e.g., full responsibility, no responsibility, primary responsibility) aligns with the legal criteria used by law enforcement officers and judges in the region. This alignment ensures that the model’s output is valid from a legal standpoint and reflects how accident liability is typically determined in practice.

Our research contributes to the field of traffic accident attribution in three significant ways: Firstly, this study proposes an approach that combines deep learning and interpretable analysis to tackle the problem of accident responsibility attribution, substantially enhancing the adaptability and accuracy of traffic accident responsibility classification. The proposed model can assist transportation departments and insurance companies in more accurately assigning accident responsibility, reducing disputes and controversies surrounding accident responsibility classification, promoting public awareness and understanding of road safety, and increasing public attention to road safety. Moreover, our research provides compelling evidence for traffic accident responsibility attribution and suggests that the AMFormer framework based on deep learning performs well in handling complex accident scenarios. Moreover, this approach is expected to be widely applied in intelligent transportation management systems, the insurance industry, and autonomous driving accident responsibility classification in the future.

Despite the significant findings of this study, several limitations should be recognized. Firstly, the dataset used in this study lacks detailed driver characteristics. For example, crucial information regarding the driver’s mental state is absent. These factors can directly influence driver behavior and affect the attribution of accident responsibility. Future research should consider incorporating more comprehensive and detailed driver characteristic data to enhance model predictive accuracy and reresponsibility. Secondly, the dataset’s description of vehicle features is incomplete [62]. Factors such as the responsiveness of braking systems, and the age and condition of the vehicle, can significantly impact accident occurrence and responsibility attribution [63]. Future research should include more complete and comprehensive vehicle characteristic data. Finally, the data used in this study is limited to the Chongqing, China region, potentially restricting the representativeness of the sample. Variations in traffic regulations, driving habits, and road conditions across different countries and regions may affect accident patterns and responsibility standards. Therefore, future studies should collect and integrate data from diverse geographical areas and accident types to construct a more generalizable and transferable accident responsibility prediction model. Based on these limitations, future research directions include: (1) expanding the dataset to incorporate more complete and detailed driver and vehicle characteristic information; (2) exploring more advanced deep learning models to improve the model’s ability to capture complex non-linear relationships; and (3) conducting cross-regional and multi-type data studies to validate the model’s generalizability and transferability.

## 6. Conclusion

This study addresses the subjectivity and inefficiency issues in current traffic accident responsibility determination by proposing a deep learning-based framework for accident responsibility prediction and attribution, namely the AMFormer-Based Framework. This framework leverages the AMFormer model to achieve accurate prediction of accident responsibility levels and enhances model transparency and result credibility through the incorporation of Shapley value-based explainable AI analysis. AMFormer-Based Framework can provide a more scientific and efficient method for accident responsibility determination for traffic management authorities and insurance companies, contributing to improved fairness and impartiality in law enforcement and promoting transparency in the responsibility attribution process. Furthermore, by revealing the key contributing factors to accident responsibility, this framework can help raise public awareness of road safety, thereby effectively preventing traffic accidents.

## Supporting information

S1 File 0702 data. (XLSX)
